# Massive Education in Prison Health in Brazil: A Look Beyond the Walls

**DOI:** 10.3390/ijerph21101350

**Published:** 2024-10-11

**Authors:** Janaina L. R. da S. Valentim, Sara Dias-Trindade, Aline de P. Dias, Alexandre R. Caitano, Laysa G. de S. Nunes, Manoel H. Romão, Felipe Fernandes, Nícolas V. R. Veras, Kelson C. Medeiros, Ronaldo S. Melo, Edneide da C. Bezerra, Antonio Quintas-Mendes, Marilyn A. A. Bonfim, Alcindo A. Ferla, Ricardo B. Ceccim, Ricardo A. M. Valentim

**Affiliations:** 1 Laboratory of Technological Innovation in Health (LAIS), Federal University of Rio Grande do Norte (UFRN), Natal 59010-090, RN, Brazil; janaina.lrsv@lais.huol.ufrn.br (J.L.R.d.S.V.); alexandre.caitano@lais.huol.ufrn.br (A.R.C.); manoel.romao@lais.huol.ufrn.br (M.H.R.); nicolas.vinicius@lais.huol.ufrn.br (N.V.R.V.); kelson.medeiros@lais.huol.ufrn.br (K.C.M.); ronaldosilvamelo77@gmail.com (R.S.M.); mel.bonfim35@gmail.com (M.A.A.B.); burgceccim@gmail.com (R.B.C.); ricardo.valentim@lais.huol.ufrn.br (R.A.M.V.); 2 Centre for Interdisciplinary Studies, University of Coimbra, 3000-186 Coimbra, Portugal; 3 Faculty of Arts, Department of History, Political and International Studies (DHEPI), University of Porto, 4150-564 Porto, Portugal; 4 Institute of Human Formation with Technologies, State University of Rio de Janeiro (UERJ), Rio de Janeiro 20550-900, RJ, Brazil; 5 Federal Institute of Education, Science and Technology of Rio Grande do Norte (IFRN), Natal 59015-000, RN, Brazil; 6 LE@D—Distance Education and eLearning Laboratory, Open University of Portugal, 1250-100 Lisbon, Portugal; 7 Oswaldo Cruz Foundation (FIOCRUZ), Rio de Janeiro 21040-360, RJ, Brazil; 8 Public Health Program, Federal University of Rio Grande do Sul (UFRGS), Porto Alegre 90620-110, RS, Brazil; ferlaalcindo@gmail.com

**Keywords:** prison system, prison health, action research, massive training, educational pathway, self-instructional learning

## Abstract

Equal access to health initiatives and services under the principles of universal and comprehensive care remains a challenge in Brazil. The realization of public health policies is further intricate when one examines the health situation of people deprived of liberty. This study showcases the “Prison System: Beyond the Walls” educational pathway, available on the Virtual Learning Environment of the Brazilian National Health System (AVASUS). The action research methodological strategy guided the pathway development, emphasizing dialogic learning. The goal was to address the need for massive training on the topic of prison health, with the model focusing on engagement through spontaneous, non-mandatory participation in the pathway courses. The pathway comprised four modules, whose educational offerings were based on the self-learning model. Students were free to choose which courses to take and in what order, as there was no prerequisite for participating in modules. Hence, students could either take all the courses or only those with which they identify their learning needs, regardless of work demands or personal interests. Structuring the pathway through action research facilitated a massive, cohesive, and continuous training process. This approach expanded knowledge and established meaningful relationships among the related topics and the key players involved: health professionals, prison officers, and people deprived of liberty. Notably, the pathway courses have surpassed the 50,000-enrollment mark, spanning all five regions of Brazil. In this context, this article presents and discusses the development of the “Prison System: Beyond the Walls” pathway, emphasizing the massive improvement of health within Brazil’s prison system and highlighting the results achieved.

## 1. Introduction

The Brazilian Constitution and the laws that establish the Brazilian National Health System (SUS) ensure health as a right for all citizens and establish this principle as a State duty [1]. However, access to health services, in light of the principles of universal and comprehensive care, remains a challenge for Brazil [2,3]. The realization of public health policies is even more complex when it comes to the health situation of people deprived of liberty since difficulties are exacerbated by confinement conditions and the social stigma linked to incarceration [4,5,6]. This scenario is further aggravated by Brazil’s prison overcrowding. As of 2023, Brazil had the third-largest incarcerated population in the world, with 811,707 people deprived of their liberty [7].

Health policy in the context of the Brazilian prison system derives from a set of state actions, which, after a decade of evaluation of the National Health Plan for the Prison System, resulted in the National Policy for Comprehensive Healthcare for People Deprived of Liberty in the Prison System (PNAISP) in 2014 [8,9,10,11,12]. This national Brazilian policy represents an important step toward guaranteeing human rights in the country. Moreover, it complies with the Criminal Enforcement Law (LEP) No. 7.210/1984 [12,13], which establishes health, educational, religious, legal, social, and material assistance for people deprived of liberty in Brazil. Therefore, the primary focus of Brazil’s Criminal Enforcement Law is to provide more favorable and humane conditions for sentence-serving [13,14].

This national policy aims to expand the actions of Brazil’s National Health System to the prison population, as it enables each prison primary care center to be viewed and integrated into the healthcare network [12,13,14,15,16]. The direct effect of the policy necessarily involves training actors within Brazil’s prison system so that programs, projects, and actions are more highly qualified and can positively and effectively impact the induction of prison health. It is, thus, essential to consider the fundamental role of the education–health dyad in inducing public health policies [17,18]. Despite Brazil’s large prison population, with more than 800,000 people incarcerated, data from the National Penitentiary Department (DEPEN) show that only 24.74% incarcerated people participate in educational activities [19], these being the ones whose right to educational assistance is guaranteed [7]. In addition to this issue, another challenge arises regarding prison health education in Brazil: the poor level of education among people deprived of their liberty. Although the illiteracy rate among prisoners is 1.76%, only 0.59% have completed higher education as of 2023 [19].

In November 2021, the Federal University of Rio Grande do Norte (UFRN), in partnership with the Brazilian Ministry of Health (MoH), launched the educational pathway for prison health, titled “Prison System: Beyond the Walls” [11,12,20]. The pathway consists of four courses, which include the triad of the Brazilian Prison System as the main target audience, namely: health professionals, prison officers, and people deprived of their liberty. The development of the pathway’s educational resources considered the triad’s uniqueness, especially with regard to people deprived of their liberty. Therefore, the resources are essentially audiovisual owing to the low literacy rates within this population. The pathway was made available on the Virtual Learning Environment of the Brazilian National Health System (AVASUS) [12,20]. AVASUS is a prominent platform in Brazil, notably for being one of the main tools of the country’s national continuing health education policy. The platform is also among the world’s most significant health education platforms, with more than 1.3 million students enrolled [21].

This study presents and discusses the development of the educational pathway, which has seen over 50,000 enrollments across Brazil’s five regions, highlighting the results in the context of large-scale continuing health education within the country’s prison system. Of note, the action research methodological strategy was adopted in this development to incorporate a dialogic learning perspective in an interactive and incremental model for producing health education courses in Brazil’s prison system.

## 2. Materials and Methods

This study employed an action research approach aimed at developing an educational pathway with technology-mediated courses for massive training. Notably, a pathway that considered the needs reported by the Brazilian prison system’s actors, who figure prominently as the prison system’s triad, i.e., prison officers, health professionals, and people deprived of liberty. As Tripp [22] explained, action research is conducted in cycles of “practice improvement”, which arise from the systematization of work in two fields: “that of practice and that of research concerning practice”.

**Figure 1:**
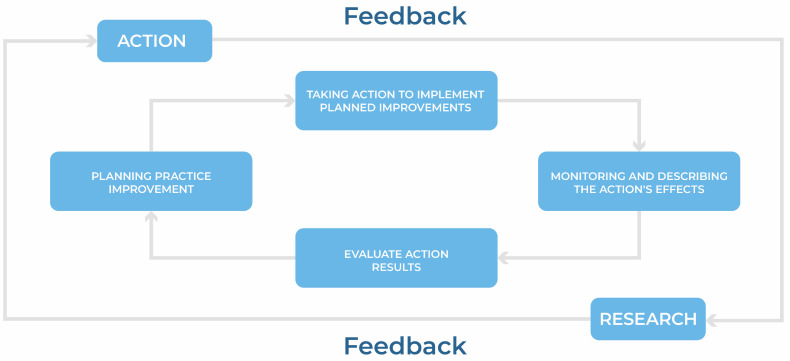
Action research model.

Figure 1 depicts the generic model of the action research process that Tripp [22] proposed, which guided the development of the “Prison System” educational pathway. Of note, the action research approach was important in building the educational pathway, as it enabled immersion in the health-related issues of the Brazilian prison system and broadened knowledge about the field. The interaction cycles of Tripp’s model [22], i.e., the actions between the stakeholders, that is, the triad of the Brazilian prison system, contributed to the development of the “Prison System” educational pathway, which is the subject and result of this study.

### 2.1. Development Flow of the Educational Pathway Based on the Action Research Methodology

**Figure 2:**
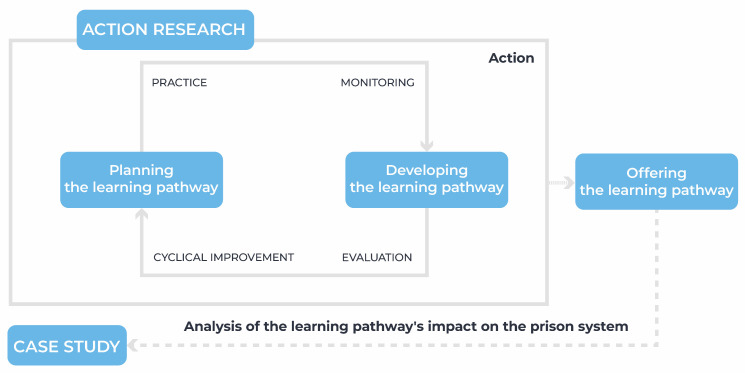
Model used to produce the “Prison System” pathway.

The action research methodology conceptualized by Tripp [22] was adapted to the context of creating the “Prison System” pathway, as Figure 2 illustrates. This was necessary because Tripp’s model [22] is a generalization. Therefore, to develop the pathway, it was necessary to include the course offering process and the case study. The impact analysis of the educational pathway is a tool for evaluating public health intervention through the training process. Validations during the course production process in the action research were carried out incrementally, i.e., at each iteration, when participants were able to evaluate and validate what was produced and suggest improvements before the course was released on AVASUS.

Importantly, the modules remained in a pilot format, for testing, in a homologation environment during the offering stage—the final stage of course production, as shown in Figure 2)—prior to their release on AVASUS. Only after validation in the homologation environment were the courses effectively made available, in a continuous flow, to students across Brazil. The course approval process was overseen by technical specialists in each area of the courses in the pathway.

According to Allen et al. [23] and Allen et al. [24], one must evaluate aspects beyond the training program in order to comprehend the impact of continuing health education. In other words, content and training quality are insufficient measures of their impact on health services. It is paramount to understand the outcomes of training and its effects on the prison system from the perspective of health professionals, prison officers, and people deprived of liberty.

In Ceccim’s view [18], to assess the impact of continuing health education, it is necessary to consider the “education–health” dyad as an area of great relevance for inducing public health policies. Thus, the action research of the educational pathway—specifically, in the post-development stage—should be evaluated from the perspective of analyzing the impacts produced on the health of the Brazilian prison system. This study presents such analyses in the Discussion section.

The development of the pathway was underpinned by an innovative workflow model to produce open educational resources for technology-mediated education. This model was developed for use within the Virtual Learning Environment of the Brazilian National Health System (AVASUS) [25]. In addition, it followed an interactive and incremental cycle, i.e., (a) practice, (b) monitoring, (c) evaluation, and (d) cyclical improvement, as Figure 2 illustrates.

**Figure 3:**
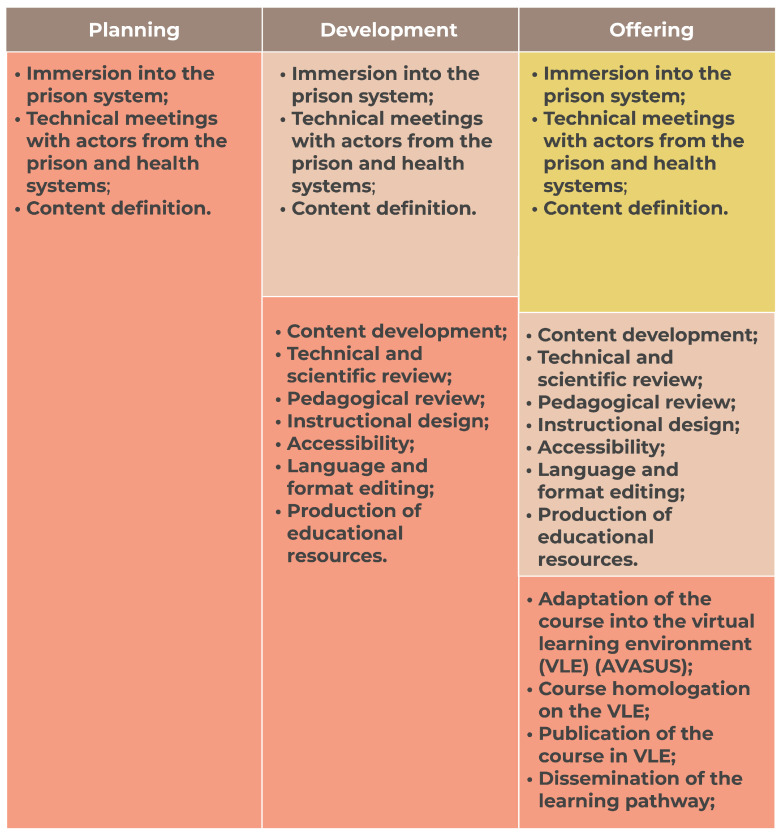
Heat map of the development of the “Prison System” pathway.

The development process was based on the continuing improvement of the content developed until it was released, according to the characteristics defined in each course’s planning. The process consisted of three stages: (i) planning, (ii) development, and (iii) offering. Each stage included a set of activities undertaken during the development process (see Figure 3). It is worth noting that each stage involved multiple participants contributing to the development of the pathway, with a total of 69 people.

In the planning stage, 28 people participated, including one individual deprived of liberty, legal judges, prison health experts, prison system experts, professors, and researchers. In the development stage, another 28 participants participated, including three people deprived of liberty, prison health experts, prison system experts, health education experts, technicians specializing in the production of open and technology-mediated educational resources, professors, and researchers. A team of 13 people participated in the offering stage—prison system experts, prison health experts, science communication technicians, and virtual learning environment technicians. As Figure 3 shows, some activities from earlier stages were repeated. However, the teams were not counted toward the total number for each stage.

A heat map was used to represent the level of effort put into each stage of the pathway development and related activities. In the representation, red means greater effort, orange means moderate, and yellow indicates light effort. The adopted process allows activities to be cumulative, making it possible to perform tasks from previous stages while reducing the intensity of current ones due to the demands imposed by a new stage.

**Figure 4:**
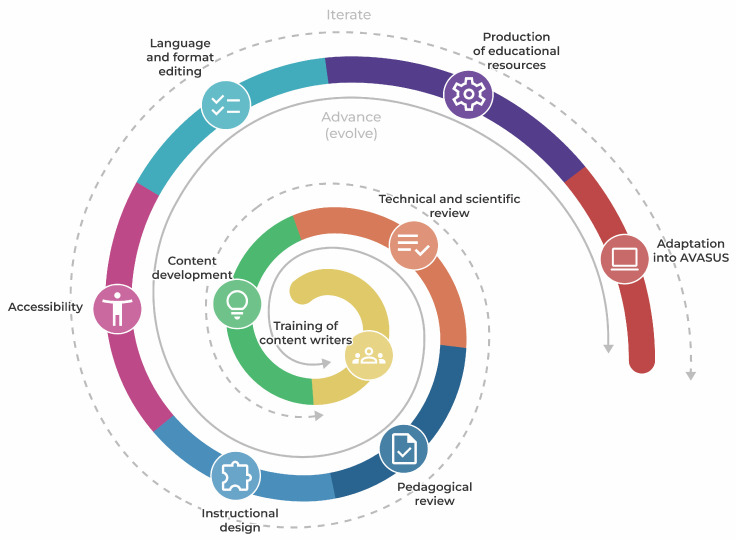
Cyclical, spiral, iterative, and incremental models.

The interaction among stage activities was required due to the continuous need for improvement in the pathway development, which occurred interactively and incrementally, following the logic of continual improvement until the course was offered. Driven by action research, the development flow of the pathway was systematized as Figure 4 illustrates.

The development flow was implemented as a process of continual improvement in the creation of the educational resources comprising the pathway. This model allowed the activities of each development stage to be conducted a certain number of times until the courses were evaluated, approved, and considered suitable for release into the Virtual Learning Environment of the Brazilian National Health System (AVASUS) [25].

The cyclical and spiraling model of continual improvement (see Figure 3 and Figure 4) offers flexibility in development for it aggregates value as it progresses toward fulfilling the requirements identified for each module. Activities can be parallel in a way that activities planned in a previous stage can be performed in a running stage. This is important because it facilitates timely detection and swift correction of errors, rather than only toward the end of the process, as typically occurs with sequential waterfall models.

Descriptions of the three stages, the activities planned for each stage, and how they were performed are presented below. For simplification purposes, it is worth noting that although activities from a previous stage can be undertaken in a current stage, they are only described in their specific stage.

#### 2.1.1. Planning

During planning, a set of activities was performed in order to grasp the prison system’s context and to organize, structure, and define the research. As Figure 3 shows, the activities carried out at this stage were as follows:

1. Prison system immersion: This is aimed at recognizing the prison system’s problems, reality, and needs. This task was accomplished through visits to prisons and other facilities related to the prison system; technical meetings with actors from the prison system and the health field; and recognition of problems and potentials.
2. Definition of priorities and the object to be developed: By recognizing the prison system context, it was possible to identify related needs, priorities, and problems. This allowed the object of the research and the priorities to be set to be more clearly defined. Given the demands of the Brazilian prison system, the criterion for choice was based on technical capacity, infrastructure, and personnel who could collaborate with the research.
3. Selection of content writers: After executing the previous activities, a team of PhDs and specialists, with experience in the fields of education, health education with technological mediation, prison system education, and prison health, discussed and defined the profile of the content writers who would be developing the “Prison System” pathway. Following this process, a public call for applications was issued for the profiles defined.
4. Training of content writers: After selecting the content writers, workshops and qualification sessions were held in order to produce technology-mediated educational resources. This was a significant task for the content writers during the planning stage, as it allowed them to gain an understanding of the workflow for educational resource production; see Figure 4. It is noteworthy that some of the writers had never produced an open educational resource before. Therefore, it was insufficient to merely have the necessary technical knowledge, as it was key to developing their competencies and skills to build dialogic educational resources that could be distributed online.
5. Content definition: After selecting and training the content writers, the team of specialists available for the production of the educational pathway was initially consolidated. It consisted of specialist researchers and specialist content writers. Henceforth, a set of meetings and discussions were conducted to define the scope of the content to be developed for the pathway, as well as to set up work strategies for the development of the modules and educational resources. Accordingly, the team of experts decided that the “Prison System” pathway would consist of four units and that the focus would primarily be on healthcare professionals, prison police officers, and people deprived of liberty—the triad of the prison system.

#### 2.1.2. Development

The development stage was structured through a set of previously planned activities aimed at content development. In this stage, participants included the following specialists: content designers, technical–scientific reviewers, pedagogical reviewers, instructional design professionals, accessibility professionals, linguistic reviewers for textual standardization, illustration and video production professionals, and website programmers. The activities in this stage are described below:

1. Content development: This task specifically covered course production by content writers. While working on content development, writers prepared the respective educational module plan for the pathway. The pedagogical review team assessed the plans for the educational modules before content development. During module production, the content writers continuously interacted with such a team. Thus, the activities happened concurrently whenever necessary. Notably, the production of the educational resources included several actors from the process, such as people deprived of liberty, prison officers, and health professionals, who contributed to the discussion of specific topics.
2. Technical and scientific review: It was invariably conducted after the content writer completed content production. To be considered complete, the content had to be validated by the pedagogical review team. During this stage, the technical–scientific team technically validated all content in the educational modules. The activity involved experts in prison health and the Brazilian prison system. To be deemed complete, the content had to be validated by the pedagogical team.
3. Pedagogical review: It was developed by experts in technology-mediated education, and the aim was to intervene pedagogically in content production, given the limited experience that the content writers had in the field of education. This task was necessary to ensure that the training itinerary of the courses available in the “Prison System” pathway could act as a mediator and facilitator of the student’s learning process by using dialogic language and problem-based situations. It played a key role in the construction of the educational pathway, as it guided the content writers through the production of the modules.
4. Instructional design: Technology-mediated education encompasses the general principles of didactics, with consideration for the decisions based on the choice of method for the content presentation, i.e., how it should be delivered to students [26]. Instructional design technicians develop such a didactic process of content creation. In the case of the “Prison System” pathway, the content designers and the pedagogical review team have also participated effectively and interactively. Instructional designers have an essential role in creating a content-based training itinerary to capture the students’ attention, interest, and motivation while maximizing learning outcomes [26,27]. In this context, the challenge was choosing and proposing the instructional design that content writers had decided to adopt. It should be noted that, as most of the content designers were not professors, they had no experience in the field of education. Therefore, the design decision was made in tandem with the pedagogical review team and implemented by the instructional design technicians.
5. Accessibility: In this task, accessibility features were added to the courses and educational resources of the educational path, such as video captions. This was carried out during educational resources development.
6. Language and format editing: Based on linguistic standards, the purpose of this task was to ensure the linguistic correctness of texts prepared by the content writers. Accordingly, the review also took into account the standardization of content following the recommendations of the Brazilian Association of Technical Standards (ABNT).
7. Production of educational resources: It was developed in collaboration with the content designers, the pedagogical review team, and the team of specialists in producing audiovisual, hypermedia, and graphic content. This activity was undertaken in the final stages of the development of the educational modules, requiring technological resources and skilled personnel. Although this was the final task of the development stage, it was occasionally necessary to refer back to the pedagogical and technical–scientific reviews for validation and fine-tuning. The production of the educational resources, particularly videos, was supervised by content writers and experts in the field of health and the prison system, who ensured the technical content guidelines were followed during the production of audiovisual materials.

#### Offering

This stage aimed at launching and providing the “Prison System” pathway in the VLE, that is, the Virtual Environment of the Brazilian National Health System (AVASUS). The “offering” stage marks the end of the development process. Nonetheless, a set of previous activities was necessary until the pathway offering was fully consolidated on AVASUS. These included:

1. Adaptation of the course into AVASUS: This activity was carried out by a technician who specializes in VLEs and was responsible for implementing the educational modules in AVASUS. Modules were previously validated by the content writers and the pedagogical and technical–scientific teams. The adaptation process adhered specifically to what was determined in the instructional design of the courses.
2. Course homologation on the VLE: After implementation and prior validation, the modules were reviewed by the technical–scientific, and pedagogical teams. The homologation stage was important in ensuring the technical and pedagogical compliance of the courses in the pathway before their release on AVASUS.
3. Publication of the course in the VLE: The publication in AVASUS was carried out by a technician who specializes in VLEs. This occurred only after homologation by the technical–scientific and pedagogical teams.
4. Dissemination of the educational pathway: After the AVASUS publication, it was still necessary to carry out the dissemination activities. The main purpose of promoting the educational pathway was to increase enrollment nationwide. As enrollment in the courses offered on the educational pathway was not mandatory, i.e., participation was completely spontaneous, it was necessary to adopt some outreach strategies. Examples include (a) publishing online articles on websites and in the press, digital social networks, and messaging apps; (b) sending direct mailings via email; and (c) holding seminars about the educational pathway.

This study did not involve personal data, samples, experimental procedures, interventions, or any personally identifiable data from human subjects. The data used are purely analytical and public, coming from the AVASUS platform transparency portal [28], and does not include any form of identification, interaction, or data collection from human participants. Therefore, as stated in resolution nos. 510/2016 [29] 674/2022 [30] of the National Health Council (CNS, acronym in Portuguese) of the Brazilian Ministry of Health, this research is exempt from registration with the Research Ethics Committee (CEP, acronym in Portuguese)/Brazil.

## 3. Results

This section describes the results achieved through the methodology used in planning, developing, and offering the “Prison System” pathway via action research. Importantly, the pathway does not have any prerequisites for enrolling in the educational modules offered. This means students are free to decide which courses to enroll in and in what order to take them. This way they can participate in all the modules of the pathway or only those they need or are interested in.

### The “Prison System: Beyond the Walls” Pathway: Pedagogical Architecture, Audience, Educational Dimensions, Massive Offering, and Student Profiles

**Figure 5:**
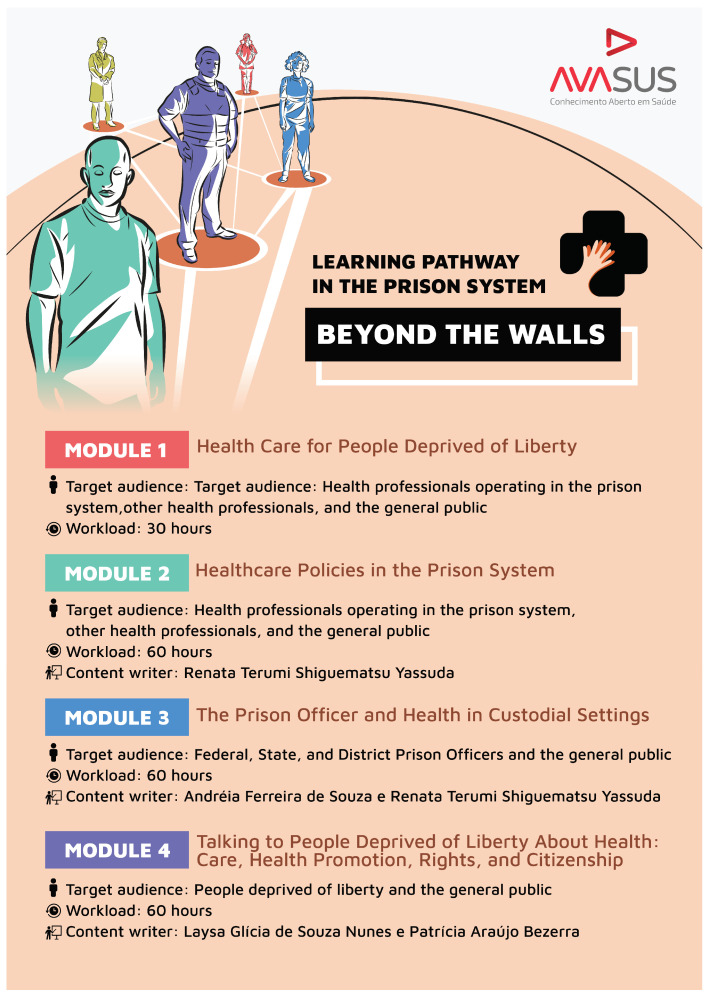
Educational pathway: modules, target audience, and workload.

The “Prison System” pathway provides a sequence of educational offers based on a self-instructional learning or self-learning model, comprising four modules. Figure 5 summarizes the courses and their respective outlines, accessible for reference at the following link: https://avasus.ufrn.br/local/avasplugin/cursos/prisional.php (accessed on 14 March 2024) [31]. In addition, it provides further details on each course, such as workload, target audience, assessments, tutoring options, launch date, objectives, and content.

Figure 5 shows how the pathway was structured, with content organized according to the training needs of the actors involved, as identified within the prison health context. As previously discussed, the pathway’s target audience was the prison system triad: health professionals, prison officers, and people deprived of liberty. The latter group accessed the educational pathway via AVASUS from prison classrooms, using prison computers. It is worth noting that there are incarcerated individuals under home detention in Brazil, who can also access the courses from home or work settings, provided they have the necessary authorization.

**Figure 6:**
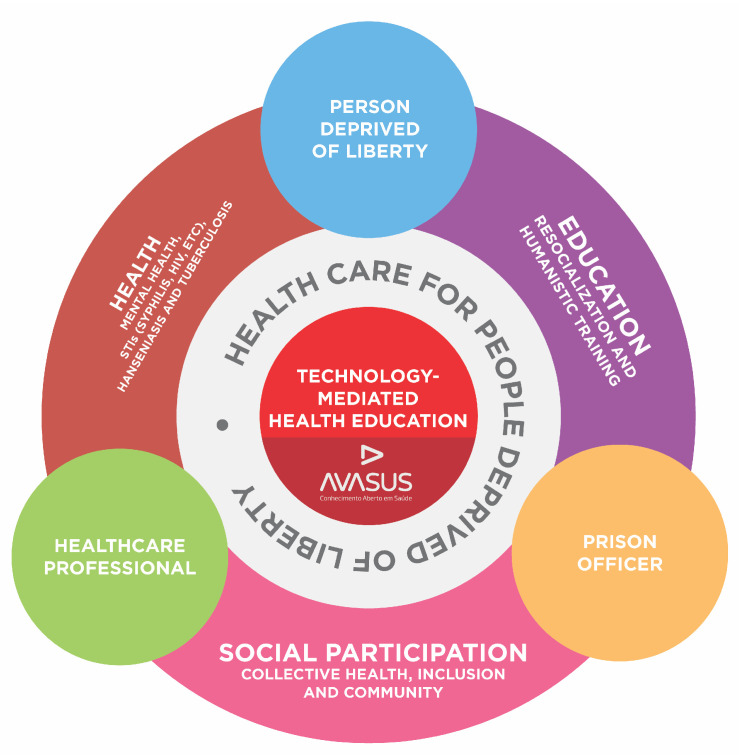
Pedagogical architecture of the “Prison System: beyond the walls” pathway.

As Figure 5 also shows, the “Prison System” pathway consisted of a 30-h introductory course and three 60-h modules, totaling 210 h of free and open courses. Transversely to the triad, the pathway also covers the following training dimensions (see Figure 6):

- Health: This dimension addresses recurring issues within the prison system, such as mental health and the most prevalent diseases in prison settings, including sexually transmitted infections (syphilis, HIV, and other STIs), tuberculosis, and leprosy. Aspects related to men’s health, women’s health, and mother and child issues in prison settings were also covered.
- Education: The aim is to provide people deprived of liberty with knowledge of their rights and duties, encouraging them to recognize and understand that health and education are rights guaranteed by the Brazilian Criminal Enforcement Law. This dimension encompasses the entire prison health triad, since, apart from people deprived of liberty, it is important to promote knowledge that can raise the awareness of all the players involved, as well as society, regarding the prison system.
- Social participation: Apart from including the prison system triad, the educational pathway has been made available to the general public, who might be interested in the subject. This includes students from the fields of health and law, prison personnel, and relatives of those deprived of liberty. In this context, it was deemed crucial that all these individuals had indiscriminate access to the courses offered. That is because it is necessary to disseminate to society that addressing prison health also implies addressing community health, thereby achieving collective and social results that transcend prison walls.

Figure 6 systematically provides the pedagogical architecture of the educational pathway and all its training dimensions, visually highlighting all the educational dimensions.

The “Prison System: Beyond the Walls” pathway has four educational modules. The first module to be offered is called “Healthcare for People Deprived of Liberty”. It characterizes the prison population and public health policies aimed at this group. Moreover, it fosters in the student the necessary reflections and knowledge on the prison system topic, with a particular focus on primary healthcare. The module was adapted from the Family Health Strategy Graduate Course, a national program offered through the More Doctors Program by the Brazilian Ministry of Health [32].

“Healthcare Policies in the Prison System” was the second course offered in the pathway. It aims to train health professionals in prison primary care teams to promote and protect the health of people deprived of liberty, in line with the National Policy for Comprehensive Healthcare for People Deprived of Liberty in the Prison System (PNAISP). This course covers several aspects of this nationwide Brazilian public policy, emphasizing human rights and health.

The third course offered was “The Prison Officer and Health in Custodial Settings,” which aims to develop skills in health-related aspects inherent to the prison officer’s role, as well as quality of life at the workplace. The primary axis of this course fostered an understanding of the ethical aspects and culture of humanization in prison settings. It also explored issues related to the prison officer’s mental health, contributing to the development of the competencies to promote care for one’s own health and the prison population’s health.

**Figure 7:**
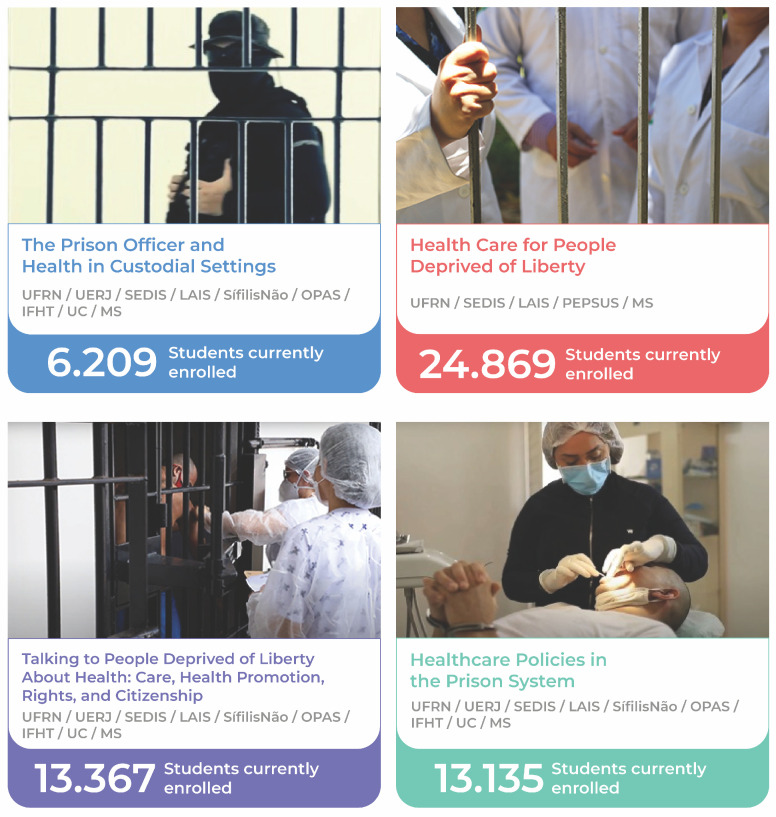
Number of students enrolling and completing the courses in Brazil as of May 2024.

The fourth course, “Talking to People Deprived of Liberty About Health: Care, Health Promotion, Rights, and Citizenship,” aimed to develop skills for health promotion and access in environments of deprivation or restriction of liberty. The primary guiding axis of the course is Brazil’s National Health System (SUS), its principles, and how it is adopted in the prison system. The “Prison System: Beyond the Walls” pathway was launched on 26 November 2021 (see Figure 5). On 12 May 2024, it already had more than 50,445 students completing courses across Brazil (see Figure 7).

Figure 7 shows the “Healthcare for People Deprived of Liberty” course features the largest number of students enrolled. This can be explained by the fact that it was the first course offered, and unlike the other three courses, it did not undergo the same development process. Instead, it was adapted for the educational pathway, thereby requiring less effort. According to Sidrim [33], the adaptation process demands less effort than creating a new course. For “Healthcare for Persons Deprived of Liberty”, only specific updates and adaptations were carried out for its release on the Virtual Learning Environment of the Brazilian National Health System (AVASUS). These were necessary for its inclusion in the educational pathway.

Of note, Figure 7 shows that, although the other three courses were launched simultaneously, enrollment numbers varied. This can be explained by the number of health professionals in Brazil, considering the different professions that form a multi-professional team. There are more than 3,000,000 health professionals [34] in contrast to 79,202 prison officers [35].

Therefore, the courses closely linked to health have higher enrollment rates. Another factor that helps explain this phenomenon is that enrollment in the pathway courses is not mandatory, as we discussed earlier regarding the architecture of the pathway. Therefore, students are free to choose, according to their interests, in which courses to enroll, meaning that there is not a uniform number of enrollments.

**Figure 8:**
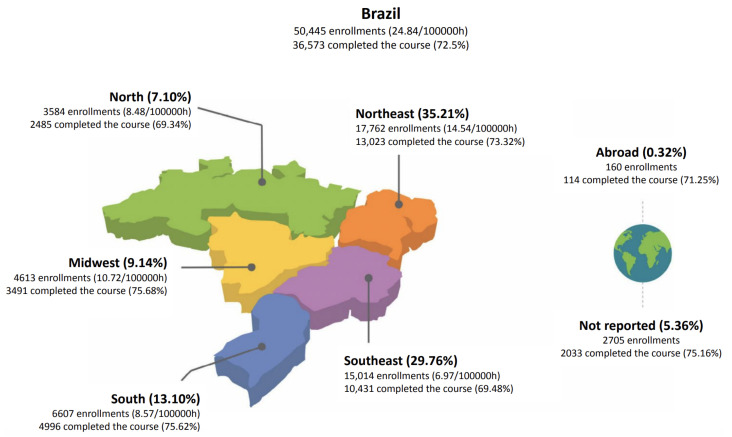
Map of enrollment distribution across Brazil.

Figure 8 presents the number of students enrolling and completing the courses both in Brazil and per country region. The map also gives the number of professionals who have enrolled, as well as the number of students without formal ties. Individuals who are not registered in the National Registry of Healthcare Facilities (CNES) of Brazil’s Ministry of Health are considered not to have formal ties. Therefore, they may be health students and others, professionals from several fields, such as law, and people from the general public with an interest in the topic.

As of 12 May 2024, a total of 50,445 students had enrolled in the pathway in Brazil. It is worth noting that 160 students enrolled from outside Brazil. These enrollments represent 0.31% of the pathway’s overall enrollments. A remarkable aspect of this statistic is that all the courses in the pathway are offered only in Brazilian Portuguese.

Another important fact to note in Figure 8 regards the Northeast region, which recorded 17,762 enrollments, and the Southeast region, which recorded 15,014 enrollments. Combined, they totaled 32,776 enrollments in the pathway courses. This means that students from both regions alone amounted to 64.97% of all enrollments. South Brazil accounted for 13.09% of enrollments in the pathway. North and Midwest Brazil represented 16.24% of enrollments. Finally, 5.70% originated from users abroad or from locations whose source of access could not be identified.

**Figure 9:**
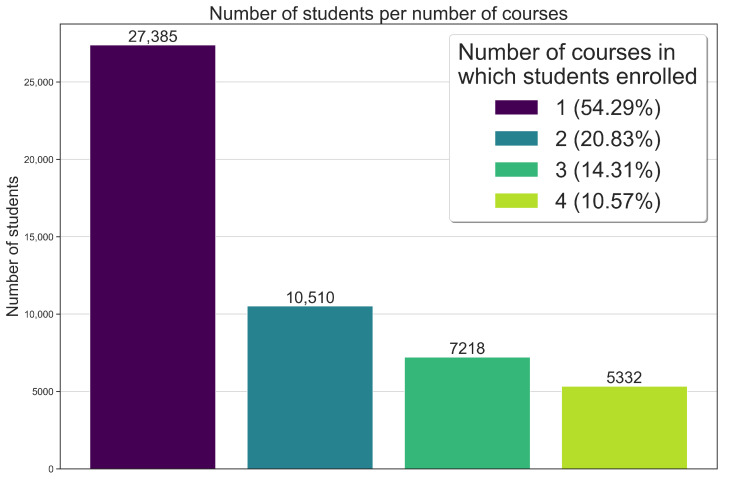
Histogram of student enrollment in courses.

Figure 9 provides an analysis showcasing how the massive training process was impacted by the pedagogical architecture, also evidencing more than 50,000 enrollments in the pathway. The results in Figure 9 reveal that the students mostly chose just one course in the pathway. It should be noted that approximately 16,000 (78.67%) students out of a total of 20,337 completed only one course. Interestingly, the number of students who took two or more courses significantly decreased, with a reduction ratio of approximately 43–50%, respectively. From two to three courses, there was a 43.03% drop, and from three to four courses, there was a 50.49% drop, as Figure 9 shows.

The findings indicate that the pathway focused on health in prison settings and developed through the action research methodology has spontaneously achieved national scalability, as demonstrated by the number of students enrolled nationwide.

## 4. Discussion

To grasp the relevance of this educational pathway to Brazil’s prison health system, it is paramount to observe and take into account the country’s particularities. This is especially due to its continental dimensions, which encompass aspects such as the vast territorial extension, population density, economic status, demographics, and cultural diversity. In 2024, Brazil’s population already stands at 220 million, making it the sixth-largest in the world. Occupying 8.5 million square kilometers, Brazil is divided into five geopolitical regions—North, Northeast, Midwest, Southeast, and South—twenty-six states, and a federal district, which is home to the country’s capital, Brasília, as well as 5570 municipalities.

In this study, it is important to consider the number of health professionals, which exceeds three million workers [34], and the size of the country’s prison population, which is approximately 750,000 individuals. The latter aspect makes Brazil the third-largest prison population globally [36].

In 1988, after the end of the military dictatorship, the current Brazilian Constitution was promulgated, establishing the internationally recognized Brazil’s National Health System (SUS, for its acronym in Portuguese). SUS has the following organizational principles: universal access, decentralized management, comprehensive care, and social participation, all defined by the Brazilian health movement [34,37].

Brazil’s National Health System is notable for being the main provider of care for 75% of the country’s population. Since its start, health system management has been decentralized to state and municipal governments. Afterward, Health Regions were created to group and integrate adjacent municipalities, as well as to better organize the planning and adequate provision of actions and health services [34,37].

Considering all these aspects, it is clear that implementing Brazil’s National Health System and its public health policies is a strenuous and intricate task. Accordingly, it demands strategic, administrative, and logical planning to meet public health needs more effectively in a country with continental characteristics. Evidently, this applies to prison health, as it also falls under the remit of Brazil’s National Health System (SUS) to guarantee healthcare for the prison population, which is considered to be in a vulnerable situation.

To discuss health in the prison context, it is essential to recognize that in Brazil, as in other nations, the incarcerated population is ever-increasing [38], which makes the prison system and prisons a major public health issue [39]. This population is vulnerable to the transmission of many diseases, such as tuberculosis, hepatitis, HIV, and syphilis [6,40,41,42,43]. In this context, the educational pathway, which is the subject of this study, contributes as a tool for inducing public health policies within Brazil’s prison system, an aspect that can be replicated in countries sharing similar characteristics and problems as Brazil.

Health promotion within the prison system also acts as an inducer of health in society; therefore, the impacts transcend the prison walls, reaching the whole community [44]. For that reason, promoting prison health has been one of the goals of healthcare in the global context [39]. Unfortunately, this remains neglected by many countries with characteristics similar to those of Brazil, including not only low- and middle-income nations [7]. According to the 2020 report “Mass Incarceration” by the Prison Policy Initiative, the prison model adopted in the United States does not facilitate possibilities for (re)socialization. This is closely related to the aspects that favor access to education for the population deprived of their liberty in the US [7,45]. This problem is also evident in the process of health promotion within the US prison system, particularly given studies highlighting the inadequate healthcare provided to those deprived of their liberty [46,47,48].

It is necessary to comprehend that promoting prison health means promoting community health. In Brazil’s scenario, promoting prison health also implies guaranteeing the health rights of people deprived of liberty. Therefore, it is not solely a question of providing healthcare and assistance but also ensuring fundamental human rights [10,12,49]. Therefore, the educational pathway developed and offered through AVASUS contributes not only to promoting prison health but also to ensuring the right to education for those deprived of liberty. Although tacit, it remains to be remembered that health and education are both inalienable human rights and thus the State cannot refrain from guaranteeing them.

In considering the issues in the prison system and the problems related to prison health in Brazil, especially due to the country’s characteristics, it becomes clear that the challenges are significant. This is because overcoming them requires scalable actions, capable of reaching the entire national territory, with all its peculiarities, in a sustainable manner. It is, therefore, essential that the National Health Policy for the Brazilian Prison System is systemically and structurally induced.

For Ceccim [17], the education–health dyad should be considered an area of knowledge of significant relevance for inducing public health policies. In the case of prison healthcare in Brazil, continuing health education must be situated, i.e., situated learning, because the healthcare scene takes place in the health service, that is, the prison system [49].

In this context, technology-mediated massive health education is proving to be a feasible path as one of the tools for inducing the national prison health policy. This finding was reinforced in this study, as the results revealed how the technological mediation promoted by the Virtual Learning Environment of the Brazilian National Health System (AVASUS) managed to massively offer the courses in the “Prison System” pathway across the Brazilian territory.

It should be noted that, due to Brazil’s peculiarities, it would be difficult to achieve the numbers recorded, which include more than 50,000 students enrolled nationwide, by relying solely on conventional models of education. It is also important to pinpoint that, due to the stigma and lack of accurate information, prison health is a topic difficult to approach.

The educational model applied in the pathway, developed based on the action research methodology, allowed the researchers to immerse themselves in the issue. This was necessary for the courses to be meaningful in the context in which the students were situated and may help to explain the students’ uptake of the courses in the pathway [12].

In line with this, the data show that enrollment in the courses along the pathway was higher in the Northeast and Southeast regions of the country, accounting for 54.95% of enrollments. Together, these regions represent 62.96% of the country’s prison population. Meanwhile, Brazil’s Southern region, which comprises 17.78% of the country’s prison population, registered 13.64% of enrollments in the pathway. In the North and Midwest, Brazil recorded 14.61% of enrollments. Both regions have the lowest prison populations in the country, at 7.89% and 11.37%, respectively [50].

Although no statistical correlation study was conducted between enrollment and prison population variables, as this was not the focus of this study, it can be observed that the size of the prison population in these two regions, as in the others, may have influenced the demand for courses and enrollment in the pathway. This observation is due to a coincidence between the size of the prison population, the proportion of enrollees per Brazilian region, and the local demands for continuing health education in the prison system.

Based on the findings and evidence presented, it is reasonable to infer that the use of AVASUS, as a platform aimed at promoting integrated knowledge that is accessible to all, is consistent with the need for humane health training. In addition, the platform’s use was fully applicable to the context of prison health.

Notably, AVASUS was selected because it is one of the largest open educational platforms in the field of health globally, with particular emphasis on the Region of the Americas [10,51,52], where the Virtual Campus of the Pan American Health Organization is also available. The platform has over 1.2 million students registered and 3.5 million enrollments in its 400-plus courses. In addition, AVASUS has issued more than two million certificates of completion, which has certainly contributed to the dissemination of the courses.

Nonetheless, the engagement and spontaneous, i.e., non-mandatory participation in the courses of the “Prison System” pathway can be explained by its development model and pedagogical architecture. The phenomenon has been attributed to the meticulous design and implementation of the program, which is based on action research principles. The receptivity observed by the path’s beneficiaries is clear proof of the effectiveness and relevance of its pedagogical structure. This shows the transformative power of health education in the prison system.

As discussed, the research conducted yielded substantial results, especially when considering the scope of the educational pathway courses. It is important, however, to report the primary limitation of the study, which pertains to the pedagogical architecture of the pathway. In its first version, the pathway focused on the prison system triad—health professionals, prison officers, and people deprived of liberty—thereby excluding prison managers from the training process. Therefore, this is a topic that should be considered in future research.

## 5. Conclusions

The development of the pathway “Prison System: Beyond the Walls” resulted in a number of lessons learned, three of which are worth highlighting. The first is the potential for the creation of high-quality content using the action research methodology in the field of health education. The second lesson, derived from the first, lies in the need to immerse oneself in the healthcare scenario, in this case in Brazil’s prison system. Interacting with prison system actors before undertaking the course development was very enriching for knowledge production. Learning about the realities imposed on the Brazilian prison system and, consequently, on people deprived of liberty, was crucial in ensuring that the content could dialog with students’ needs and experiences. This way the courses and their content could, in effect, have a dialogic architecture. The third lesson learned, derived from the second, was recognizing the importance of including prison officers in all stages of knowledge production. Therefore, to achieve engagement and involvement in a way that extends beyond access to the prison system, it is necessary to value and include these actors in the process of knowledge creation. Prison officers are essential pillars of the Brazilian prison system; without them, nothing could conceivably succeed.

These lessons learned have equipped AVASUS to implement a massive health training process within Brazil’s National Health System, owing to the technological mediation that has enabled the training to reach a large scale throughout Brazil. This success is partly because immersion in the prison system has allowed courses to align content with the daily realities of the prison environment, making the material more relevant to the students. AVASUS has also contributed to positive impacts on health services, demonstrating that massive training using this technology has fostered resilience and responsiveness in Brazil’s health system. As the results indicate, students reported that the training enabled them to change their work practices, which was a positive factor in improving healthcare and assistance for people deprived of their liberty.

During crises, the potency of AVASUS was observed, such as during the syphilis epidemic and the COVID-19 pandemic, which occurred simultaneously in Brazil [10,20,21,53,54]. Therefore, AVASUS is proving to be a robust tool for inducing public health policies, as is the case with prison health in Brazil, through the implementation of strategies for massive and continuing health education [52].

The pedagogical architecture of the pathway was structured according to the training needs identified in the prison health context through action research. In this way, the organization of the pathway allowed for a massive, cohesive, and continuing training trajectory. It occurred through the deepening of knowledge and establishment of meaningful relationships between the related topics and the main actors involved: health professionals, prison officers, and people deprived of liberty.

The use of the self-learning model through the technological mediation of the pathway is also shown to promote health system resilience [21,54,55]. In the specific case of the “Prison System” pathway, the model facilitated the promotion of health beyond the walls of Brazilian penitentiaries. Thus, it can be argued that the action research methodology promoted the development of an educational pathway for prison health, which spontaneously achieved national scalability, as demonstrated by the number of enrollees in the pathway from all parts of the country.

Using the action research methodology, it is possible to structure and produce an educational pathway focused on prison health and, thereby, achieve national scalability with engagement through spontaneous, non-mandatory adherence. Hence, it is possible that the methodology chosen could also be replicated in other public health contexts. This is the case not only for Brazil but also for other countries that need to scale up the process of continuing education for their healthcare workers, in view of the needs imposed by their health systems and public health policies.

As a prospect for future research, it is essential to emphasize the need to apply action research to the development of educational courses and resources for training prison managers. This aspect would involve an additional actor in the educational pathway, which would incorporate not only the triad in its pedagogical architecture but also the quadrilateral of the Brazilian prison system, namely, health professionals, prison officers, people deprived of liberty, and prison managers.

## Data Availability

Data are contained within the article.
